# Genome Sequences of Beak and Feather Disease Virus in Urban Rainbow Lorikeets (*Trichoglossus haematodus*)

**DOI:** 10.1128/genomeA.00283-15

**Published:** 2015-04-23

**Authors:** David A. Collings, Berwyn G. Collings, Laurel Julian, Brigitta Kurenbach, Arvind Varsani

**Affiliations:** a School of Biological Sciences and Biomolecular Interaction Centre, University of Canterbury, Christchurch, New Zealand; b Turramurra, NSW, Australia; c Department of Clinical Laboratory Sciences, Electron Microscope Unit, Division of Medical Biochemistry, University of Cape Town, Observatory, South Africa; d Department of Plant Pathology and Emerging Pathogens Institute, University of Florida, Gainesville, Florida, USA

## Abstract

Beak and feather disease viral genomes were recovered from two deceased juvenile urban rainbow lorikeets (*Trichoglossus haematodus*) that lacked tail feathers. These genomes share ~95% pairwise identity with two beak and feather disease virus (BFDV) genomes identified in wild and captive Australian *T. haematodus* birds and ~92% identity to those in wild New Caledonian *T. haematodus deplanchii* birds.

## GENOME ANNOUNCEMENT

Beak and feather disease virus (BFDV) is a circular single-stranded DNA virus belonging to the *Circoviridae* family. The BFDV genome is ~2 kb and encapsidated into icosahedral virions (~17 to 25 nm in diameter). BFDV infects >65 parrot species and both wild and captive parrot populations, having a near-global distribution (1, 2). The bidirectionally transcribed genome of BFDV encodes at least two proteins, the replication-associated protein (Rep) transcribed from the virion strand and the capsid protein (CP) transcribed from the complementary strand.

BFDV causes beak and feather disease in parrots, the symptoms of which include depression, lethargy, diarrhea, and feather loss (3–7). BFDV can be spread through viral particles shedding in feather dust, crop secretions, and feces. In captivity, environmental contamination is probably the most prevalent route of transmission (8).

A wild urban rainbow lorikeet (*Trichoglossus haematodus*) pair nesting in a suburban garden in Sydney, Australia, initially produced nestlings in 2008 that failed to fully develop feathers. BFDV was identified from one such deceased nestling in 2009 (9). In late 2012, the adults fledged three nestlings lacking tail feathers. This was first observed in early November 2012, and these birds still lacked tails by late November. The birds had dark beaks, confirming that they were juveniles, while the parents had red beaks, a full complement of tail feathers, and no apparent disease. Flight for the juvenile birds was difficult and uncontrolled, and they preferred to climb shrubs and trees with their claws and beaks. Feathers were collected from the remains of two birds that succumbed to domestic cats, while the third nestling, alive in early January 2013 but still lacking tail feathers, disappeared soon afterward. The adult pair raised clutches in late 2013 and late 2014, in which the juveniles had complete tail feathers. However, the adult male developed feather abnormalities on his back. It seems likely, although it has not been tested, that the adult pair has survived as carriers of BFDV for at least 7 years.

Total DNA was isolated from the feather samples of the two deceased juvenile rainbow lorikeets using the Extract-N-Amp blood kits (Sigma-Aldrich, USA), as previously described (10, 11). BFDV genomes were recovered using abutting primers, cloned, and Sanger sequenced, as previously described (9, 12). The two 2,015-nucleotide (nt) BFDV genomes share 99.26% genome-wide pairwise identity. These new genomes share ~95% genome-wide pairwise identity with the only two other *T. haematodus*-associated BFDV isolates (accession numbers JX049195 and AF311299), both from Australia, one sampled in 2000 in Victoria (13), and the other in 2009 from New South Wales (9). The New Caledonia *T. haematodus deplanchii*-associated BFDV genomes share ~92% pairwise identity, and the rest of the BFDV isolates from other parrot species share 84% to 90% pairwise identity.

The genomes of BFDV identified in *Trichoglossus* spp. are most closely related to each other. Phylogenetic analysis shows that they are monophyletic, with two genotypes, one associated with *T. haematodus deplanchii* in New Caledonia, and the other with *T. haematodus* in Australia, thus suggesting host specificities of these strains of BFDV.

### Nucleotide sequence accession numbers. 

The complete genomes of BFDV have been deposited at GenBank under the accession numbers KP795105 and KP795106.
